# Neurotensin-polyplex-mediated brain-derived neurotrophic factor gene delivery into nigral dopamine neurons prevents nigrostriatal degeneration in a rat model of early Parkinson’s disease

**DOI:** 10.1186/s12929-015-0166-7

**Published:** 2015-07-22

**Authors:** Nancy G. Hernandez-Chan, Michael J. Bannon, Carlos E. Orozco-Barrios, Lourdes Escobedo, Sergio Zamudio, Fidel De la Cruz, Jose L. Gongora-Alfaro, Juan Armendáriz-Borunda, David Reyes-Corona, Armando J. Espadas-Alvarez, Yazmin M. Flores-Martínez, Jose Ayala-Davila, Maria E. Hernandez-Gutierrez, Lenin Pavón, Refugio García-Villegas, Rasajna Nadella, Daniel Martinez-Fong

**Affiliations:** Departamento de Fisiología, Biofísica y Neurociencias, CINVESTAV, Av. Instituto Politécnico Nacional # 2508, México D.F, 07360 Mexico; Department of Pharmacology, Wayne State University School of Medicine, 540 E Canfield Ave, 3355 Scott Hall, Detroit, MI 48201 USA; Escuela Nacional de Ciencias Biológicas, IPN, Wilfrido Massieu s/n, México D.F, 07738 Mexico; Departamento de Neurociencias, Centro de Investigaciones Regionales “Dr. Hideyo Noguchi”, Universidad de Yucatán, Av. Itzaes # 490 x 59-A, Mérida, Yucatán 97000 Mexico; Institute for Molecular Biology and Gene Therapy, Department of Molecular Biology and Genomics, University of Guadalajara, Sierra Mojada # 950, Guadalajara, Jalisco 44281 Mexico; Department of Psychoimmunology, National Institute of Psychiatry “Ramón de la Fuente”, Calzada México Xochimilco # 101, México D.F, 14370 Mexico; Program de doctorado en Nanociencias and Nanotecnología, CINVESTAV, Av. Instituto Politécnico Nacional # 2508, México D.F, 07360 Mexico

**Keywords:** Neurorestoration, Neurodegeneration, Parkinson’s disease, Gene therapy, Neurotrophic therapy

## Abstract

**Background:**

The neurotrophin Brain-Derived Neurotrophic Factor (BDNF) influences nigral dopaminergic neurons via autocrine and paracrine mechanisms. The reduction of BDNF expression in Parkinson’s disease substantia nigra (SN) might contribute to the death of dopaminergic neurons because inhibiting BDNF expression in the SN causes parkinsonism in the rat. This study aimed to demonstrate that increasing BDNF expression in dopaminergic neurons of rats with one week of 6-hydroxydopamine lesion recovers from parkinsonism. The plasmids phDAT-BDNF-flag and phDAT-EGFP, coding for enhanced green fluorescent protein, were transfected using neurotensin (NTS)-polyplex, which enables delivery of genes into the dopaminergic neurons via neurotensin-receptor type 1 (NTSR1) internalization.

**Results:**

Two weeks after transfections, RT-PCR and immunofluorescence techniques showed that the residual dopaminergic neurons retain NTSR1 expression and susceptibility to be transfected by the NTS-polyplex. phDAT-BDNF-flag transfection did not increase dopaminergic neurons, but caused 7-fold increase in dopamine fibers within the SN and 5-fold increase in innervation and dopamine levels in the striatum. These neurotrophic effects were accompanied by a significant improvement in motor behavior.

**Conclusions:**

NTS-polyplex-mediated BDNF overexpression in dopaminergic neurons has proven to be effective to remit hemiparkinsonism in the rat. This BDNF gene therapy might be helpful in the early stage of Parkinson’s disease.

## Background

The brain-derived neurotrophic factor (BDNF) is a member of the neurotrophin family that is expressed by dopamine (DA) neurons along with its high-affinity receptor TrkB (tropomyosin related kinase B) [1, 2]. The expression of BDNF and TrkB during the prenatal and early postnatal development is crucial for the induction of the DA phenotype, determination of the number of mesencephalic DA neurons, and the formation of synaptic connectivity in the target innervation nuclei [3, 4]. In the adult brain, BDNF promotes neuron survival and protection of nigral DA neurons against toxic insults [5–7]. Accordingly, inhibiting BDNF expression by antisense oligonucleotide infusion causes loss of nigral DA neurons and mimics parkinsonism in adult rats [8]. In Parkinson’s disease (PD), the significant reduction of BDNF and TrkB in the substantia nigra (SN) might contribute to the death of DA neurons thus worsening the neurodegeneration [9–11]. Therefore, reinforcing BDNF expression in DA neurons might rescue the nigrostriatal DA pathway from neurodegeneration.

To date, protective effects of BDNF have been shown in animals using procedures that supply BDNF into the striatum before the lesion. For instance, by infusion of BDNF protein [12], grafts of fibroblasts or astrocytes genetically engineered to produce BDNF [13, 14], or gene-therapy approaches using viral vectors [15]. Nevertheless, the delivery of BDNF into the striatum is inefficient to cause restorative effects in chronic neurodegeneration due to the scarcity of the nigrostriatal terminals, limiting its retrograde transport to the nigral cell bodies [16, 17] where is needed to provide neuron survival and stimulate the synthesis of proteins for the growth of neurites and axons [18]. The restorative effect of BDNF delivery into the SN on degenerated nigrostriatal DA pathway remains controversial. A study using human mesenchymal stem cells epigenetically modified to secrete BDNF grafted in the SN one week after the 6-hydroxydopamine (6-OHDA) lesion causes significant hypertrophy of nigral DA neurons, increase of striatal innervation and stabilization of amphetamine-induced motor activity [19]. In contrast, a study using an adeno-associated viral (AAV) vector to transduce BDNF in the SN of hemiparkinsonian rats showed no neurotrophic effects on DA neurons and striatal innervation, although blocked their motor asymmetries induced by amphetamine [20].

The neurotensin (NTS)-polyplex is a promising alternative to viral vectors to be used in gene therapy for PD [21]. The NTS-polyplex consists of nanoparticles resulting from compaction of a plasmid DNA (pDNA) by the electrostatic binding of a karyophilic peptide (KP) and the NTS-carrier, which is a conjugate of poly-L-lysine, NTS and a fusogenic peptide (FP) [22–24]. The NTS carrier enables the delivery of genetic cargo to DA neurons via NTS receptor type 1 (NTSR1) internalization [25–28]. The inclusion of the human DA-transporter (hDAT) gene promoter in the NTS-polyplex provides a second point of selectivity to transgene expression in vivo [21, 24]. In a rat model of PD, the intranigral transfection of NTS-polyplex harboring the human glial-cell derived neurotrophic factor (hGDNF) gene caused structural and functional restoration of nigrostriatal pathway that correlated with the hemiparkinsonism remission [29]. These results indicate that the transfection of neurotrophic genes into DA neurons is the correct strategy to simultaneously affect their soma and terminals [21]. On this basis, we propose that BDNF overexpression in surviving DA neurons in the early stage of Parkinsonism will prevent the nigrostriatal deterioration. To test this hypothesis, a single dose of NTS-polyplex harboring the BDNF-flag transgene under the control of hDAT (aka SLC6A3) gene promoter sequence was injected into the SN of hemiparkinsonian rats at week one after a 6-OHDA injection into the ipsilateral striatum. We showed that in a 6-OHDA-induced early hemiparkinsonism, surviving DA neurons conserve the expression of NTSR1 on cell bodies and neurites that enabled gene transfection by NTS-polyplex. Two weeks after transfection, the residual DA neurons expressed BDNF-flag, which was accompanied by a significant sprouting of DA fibers within the SN and partial restoration of both innervation and dopamine levels in the striatum, as well as a significant improvement of motor behavior. Since residual DA neurons in PD patients still conserve levels of NTSR1 [30–32] and TrkB [10], the NTS-polyplex-mediated BDNF gene therapy might be feasible and useful in the early stage of PD.

## Methods

### Plasmids

The plasmid phDAT-BDNF-flag (10.5 kbp) coding for BDNF-flag under the control of hDAT gene promoter was obtained from cloning 868 bp of the BDNF-flag coding sequence into *Not* I-*Sal* I sites of phDAT-EGFP [24, 33].

The plasmid phDAT-EGFP (10.45 kbp) coding for enhanced green fluorescent protein (GFP) under the control of hDAT gene promoter was obtained from cloning 6250 bp of the 5′-flanking regulatory region of the hDAT promoter into *Eco47* III-*Bgl* I sites of pEGFP-N1 (Clontech; Palo Alto, CA, USA) [24, 33, 34].

### Synthesis of the neurotensin carrier and polyplexes

The detailed procedures of NTS-carrier synthesis and of NTS-polyplex formation at an optimum molar ratio are reported elsewhere [24, 27, 35]. Briefly, NTS (Sigma-Aldrich; Saint Louis, MO, USA) and FP (GLFEAIAEFIEGGWEGLIEGCAKKK; purity > 90 %; SynPep; Dublin, CA, USA) were cross-linked with poly-L-lysine (48 kDa mean molecular mass; Sigma-Aldrich; Saint Louis, MO, USA) using LC-SPDP (Thermo Scientific Pierce; Rockford, IL, USA) as the cross-linker [35]. Suitable gel-filtration chromatography was used to purify the SPDP-derivatives and the NTS-SPDP-(FP-SPDP)-poly-L-lysine conjugate, the NTS carrier. This conjugate was concentrated to 1 mL, further dialyzed against phosphate-buffered saline solution, pH 7.4 (PBS), and sterilized by filtration.

The NTS-polyplexes were made by electrostatically binding the mutant Vp1 SV40 KP (MAPTKRKGSCPGAAPNKPK; 90 % purity; Synpep Corp., Dublin, CA, USA) to pDNA [24, 27, 35]. Retardation and retention gel assays were used to determine and calculate the optimal molar ratio of NTS-polyplex components as described in detail elsewhere [22, 24, 25, 27, 35]. Accordingly, the final optimum molar ratio of NTS-polyplex components for all the plasmids used were 30 nM pDNA: 20 μM KP: 720 nM NTS-carrier and, at this molar ratio, the concentration of NTS used was 720 pmol/μL, calculated as per ^125^I-NTS [23, 24]. Based on the concentration and size of pDNAs, the total amount of pDNA injected was 624 ng/3 μL for the phDAT-BDNF-flag and 618 ng/3 μL for the phDAT-EGFP.

### Experimental animals and stereotaxic surgery

All procedures were in accordance with the Mexican current legislation, NOM-062-ZOO-1999 (SAGARPA), based on the Guide for the Care and Use of Laboratory Animals, NRC. The Institutional Animal Care and Use Committee approved our animal use procedures (protocol # 0109–02). All efforts were made to minimize animal suffering.

Deeply anesthetized male Wistar rats (body weight 210–230 g) were fixed on a stereotaxic apparatus (Stoelting; Wood Dale ILL, USA). After trepanation, 3 μL of either free base 6-OHDA (6.6 μg/μL in PBS containing 0.2 % ascorbic acid; Sigma-Aldrich; St Louis, MO) or PBS (sham lesion) were injected into the left striatum at 0.25 μL/min flow rate in the coordinates AP, 0 mm from bregma; ML, + 4 mm from midline, DV, −5.2 mm from the dura mater [36]. The transfection was made in the ipsilateral SN at the coordinates AP, − 5.4 mm from bregma; ML, + 1.5 mm from midline, DV, − 6.8 mm from dura mater [37]. Three microliters of either NTS-polyplex or DMEM (sham transfection referred to as DMEM) were injected at a flow rate of 0.1 μL/min [37].

### Reverse Transcription-Polymerase Chain Reaction (RT-PCR)

RT-PCR was used to show BDNF-flag, NTSR1, GFP, and actin (housekeeping gene) mRNA expression in the transfected SN following the extraction method and the retrotranscription protocol as described previously [37]. To amplify a 163 bp fragment of BDNF-flag, the forward primer was 5′-GCAATGCCGAACTACCCAATC-3′, and the reverse primer was 5′- CTTGTCATCGTCGTCCTTGTAGTC-3′. To amplify a 537 bp fragment of NTSR1, the forward primer was 5′-CGTAAAGACCTCTATGCCAA-3′ and the reverse primer was 5′- ACCTCCTGTTGCTGATCCAC-3′. To amplify a 608 bp fragment of GFP, the forward primer was 5′-CTGGTCGAGCTGGACGGCGAC-3′ and the reverse primer was 5′-AGAGTGATCCCGGCGGCGGTC-3′. To amplify 349 bp fragment of actin, the forward primer was 5′-CGTAAAGACCTCTATGCCAA-3′ and the reverse primer was 5′-ACTCCTGCTTGCTGATCCAC-3′. After an initial denaturation at 94 °C for 5 min, amplification was made with 35 cycles for BDNF-flag, GFP and actin and 40 cycles for NTSR1 as follows: denaturation, 94 °C for 1 min, annealing, 60 °C for NTSR1 and actin, 63 °C for BDNF-Flag or 66 °C for GFP; extension, 72 °C for 30 s for BDNF-Flag, 36 s for GFP and actin and 45 s for NTSR1. PCR products were analyzed by 1.5 % agarose gel or 5 % polyacrylamide gel (BDNF-flag) electrophoresis, stained with ethidium bromide, and photographed with a Kodak DC290 camera. Amplification of their respective plasmids at same conditions was maintained as a positive control.

### Immunostaining

The presence of the NTSR1 and expression of the BDNF-flag or GFP in tyrosine hydroxylase (TH) + nigral neurons were shown by double immunofluorescence techniques using the procedure described elsewhere [24, 29]. The primary antibodies were a rabbit anti-TH (1:300 dilution; Chemicon; Temecula, CA), a mouse anti-flag (1:400 dilution; Sigma-Aldrich; St. Louis, MO, USA) and a mouse anti-GFP (1:400; Sigma-Aldrich; St. Louis, MO). The secondary antibodies were a goat anti-rabbit FITC (1:400 dilution; Jackson ImmunoResearch Laboratories Inc.; West Grove, PA, USA) and a donkey anti-mouse TRITC (1:400 dilution; Jackson ImmunoResearch Laboratories Inc.; West Grove, PA, USA). The detection of the NTSR1 in TH+ neurons was made using a mouse monoclonal anti-TH (1:400; Sigma-Aldrich; St. Louis, MO, USA) and a goat polyclonal anti-NTSR1 (1:400; Sigma-Aldrich; St. Louis, MO, USA). The secondary antibodies were a donkey anti-mouse IgG-FITC (1: 500; Jackson Immunoresearch; West Grove, PA, USA) and a Texas red rabbit anti-goat IgG (1:500; Vector Laboratories; Burlingame, CA, USA). For qualitative illustrations of transgene expression, fluorescence labeling was detected by a multispectral confocal laser-scanning microscope (TCS-SPE, Leica; Heidelberg, Germany) at excitation-emission wavelengths of 488–522 nm (green channel) and 568–635 nm (red channel). For quantitative analysis, a Leica DMIRE2 microscope was used to detect the fluorescence at excitation-emission wavelengths of 488–522 nm (green for FITC) and 568–585 nm (red for TRITC and Texas red) and the immunohistochemical labeling with bright field illumination. The images were digitalized with a Leica DC300F camera (Nussloch, Germany) and processed using the v.1.46r ImageJ program (National Institutes of Health; Bethesda, MD) in the automatic counting mode. Transfection efficiency was expressed as percentage of flag or GFP expression with respect to the total TH+ cells (*n* = 3 rats per each experimental condition). TH-immunohistochemistry was made in 10 slices taken every 120 μm from SN and striatum per rat (*n* = 4 rats per experimental group) using a monoclonal mouse anti-TH (1:1000; Sigma-Aldrich; St. Louis, MO, USA), a biotinylated horse anti-mouse IgG (H + L) (1:200), and a VectaStain Elite ABC kit with 3,3′-diaminobenzidine (Vector Laboratories; Mexico City, Mexico) as reported previously [29, 38]. SN slices were counterstained with Cresyl Violet as reported previously [38]. The digitalized images of TH-immunohistochemistry were processed using the v.1.46r ImageJ program. The background intensity was detected in an area without immunohistochemical staining and eliminated from the measurements. To count mostly TH-immunoreactive cells, 2 to 3 micrographs per slice were taken using a 20x objective (total = 25 to 30 micrographs of each SN per experimental condition) and only those darkly stained neurons located lateral to a line that divides the cerebral peduncle from the interpeduncular space on each side were considered [39]. The mean intensity of TH-immunoreactivity was determined in fibers and cells in the SN and only in fibers in the striatum (4 representative levels per nucleus per experimental condition; *n* = 4 rats), and expressed as pixel counts.

### HPLC analysis

DA content was determined in supernatants from homogenates of the SN or striatum using reverse-phase high pressure liquid chromatography (HPLC) and electrochemical detection, as described elsewhere [29, 38, 40]. Briefly, tissue samples were homogenized in 0.1 M HClO_4_ containing 3, 4-dihydroxybenzylamine hydrobromide (50 μg/μL) at a weight/volume ratio of 1:5 for SN and 1:10 for striatum. Homogenates were centrifuged in a Beckman Airfuge ultracentrifuge (Beckman Coulter, Inc.; Brea, CA, USA) at 10 psi for 15 min at room temperature, and then supernatants were filtered through 0.22 Millex-GV syringe filters (Merck Millipore; Mexico City, Mexico). Using a Rheodyne injector valve (Model 7125), 5 μL of filtrated supernatants were injected into a Velosep RP-18 reverse-phase column (3 μm, 100 × 3.2 mm; PerkinElmer; Waltham, MA, USA) heated at 30.5 °C. The mobile phase buffer was 25 mM NaH2PO_4_, 50 mM Na-Citrate, 0.03 mM EDTA, 10 mM diethylamine HCl, 2.2 mM Octylsulfonic acid/sodium salt (pH 3.2). One liter of the buffer was mixed with 30 mL of methanol and 22 mL of dimethylacetamide to form the mobile phase that was delivered by a BAS HPLC PM-80- Pump (Bioanalytical Systems; West Lafayette, IN, USA) in isocratic elution mode at 0.5 mL/min. The oxidation potential of the glassy carbon electrode was set by a LC-4C electrochemical detector at +0.75 V with respect to the Ag/AgCl reference electrode (Bioanalytical Systems; West Lafayette, IN, USA). Chromatograms were recorded and analyzed by using ChromGraph® 2.34.00 REPORT 2.30© software of Bioanalytical Systems, Inc. The pellets were resuspended in 120 μL of 0.1 M NaOH for protein determination using the Coomassie Plus assay kit (Pierce Biotechnology Rockford; IL, USA) as reported elsewhere [29, 38, 40]. Dopamine content was expressed as pg DA/μg protein.

### Behavioral testing

The spontaneous motor activity in a new environment was measured using a black-painted wooden square box (60-cm width and 50-cm height per wall) [41]. The control and experimental animals were placed individually in the middle of the box and their motor activity was recorded for 30 min. The motor analysis was made using a video image analyzer (Videomex-V, Columbus Instruments; Columbus, OH, USA) at 3-min intervals using the variables: distance traveled, walking time, resting time, and stereotypic movements (short repetitive nondisplacing movements such as scratching, grooming, and head shaking). To prevent the animals from learning, this test was made only once at one week after the lesion made with 6-OHDA or the sham striatal injection and at two weeks after the BDNF-flag, GFP or sham DMEM transfections. To avoid residual drug effects, the behavioral testing was made before the evaluation of the drug-generated behaviors. All behavioral assays were made during the light phase between 10 h and 14 h as reported by previous works [41–44].

The circling behavior was assessed as described previously [29, 37, 38]. Two treatments were used to generate circling behavior; 1) Intraperitoneal injections of amphetamine (8 mg/kg; diluted in 1 mL of saline solution; Sigma-Aldrich; St. Louis, MO) at 8 days and 23 days postlesion, and 2) Subcutaneous injections of apomorphine (0.5 mg/kg; diluted in 1 mL of saline solution; Sigma-Aldrich; St. Louis, MO, USA) tested at 9 days and 24 days postlesion. The circling behavior was monitored for 90 min and the records were collected automatically. Only those animals that showed ≥ 1,000 total turns in the amphetamine test were considered for transfection with the phDAT-BDNF-flag and phDAT-EGFP or sham transfection (DMEM).

### Statistical analysis

All values are expressed as the mean ± SE. The statistical differences were analyzed using GraphPad Prism 4 (San Diego, CA, USA). A one-way ANOVA and Bonferroni’s multiple comparisons post hoc test were used to compare the open field and cell counting results from different treatments. A nonparametric Kruskal-Wallis test and Dunns multiple comparison post hoc test were used to compare the results for amphetamine and apomorphine among all the groups. The accepted significance was at *P* < 0.05.

## Results

### Residual DA neurons conserve NTSR1 in early hemiparkinsonism

**Fig. 1:**
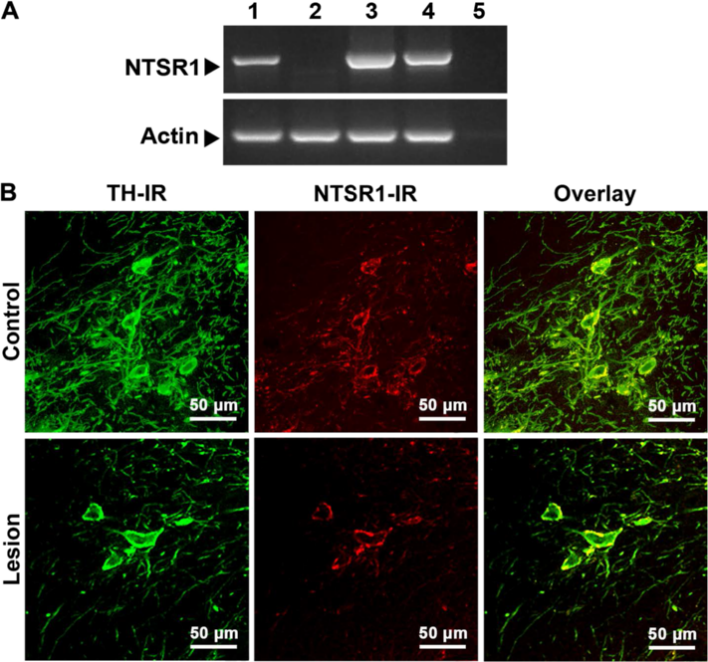
NTSR1 mRNA and protein expression in the rat substantia nigra at the day of transfection (one week after a unilateral striatal 6-OHDA lesion). **a** Representative photograph of RT-PCR amplicons showing NTSR1 expression. Actin was used as a control. Lane 1, N1E-115 cells (positive control); lane 2, L929 cells (negative control); lane 3, intact SN (substantia nigra); lane 4, SN with lesion; lane 5, PCR reagents without cDNA (internal control). **b** Representative confocal micrographs showing TH-immunoreactivity (IR) and NTSR1-IR in the mesencephalon of a hemiparkinsonian rat. The control micrographs correspond to the intact SN contralateral to the side with the lesion

One week after the 6-OHDA lesion (early hemiparkinsonism), a reverse transcription-polymerase chain reaction (RT-PCR) analysis showed the expression of NTSR1 mRNA in the SN with 6-OHDA lesion and in the positive controls (intact SN and N1E-115 cells), but not in the negative controls (L929 cells and PCR reagents without cDNA) (Fig. 1a). A confocal microscopy analysis showed that NTSR1 immunoreactivity colocalized with TH+ neuritric processes and cell bodies in the intact SN (Fig. 1b). One week after the 6-OHDA lesion, the number of neuritric processes and cell bodies with double immunoreactivity to TH and NTSR1 showed a significant decrease in the SN (Fig. 1b). These results indicate that surviving DA neurons maintain the expression of NTSR1 protein thus facilitating the transfection of transgene mediated by NTS-polyplex.

### NTS-polyplex transfects DA neurons in early hemiparkinsonism

**Fig. 2:**
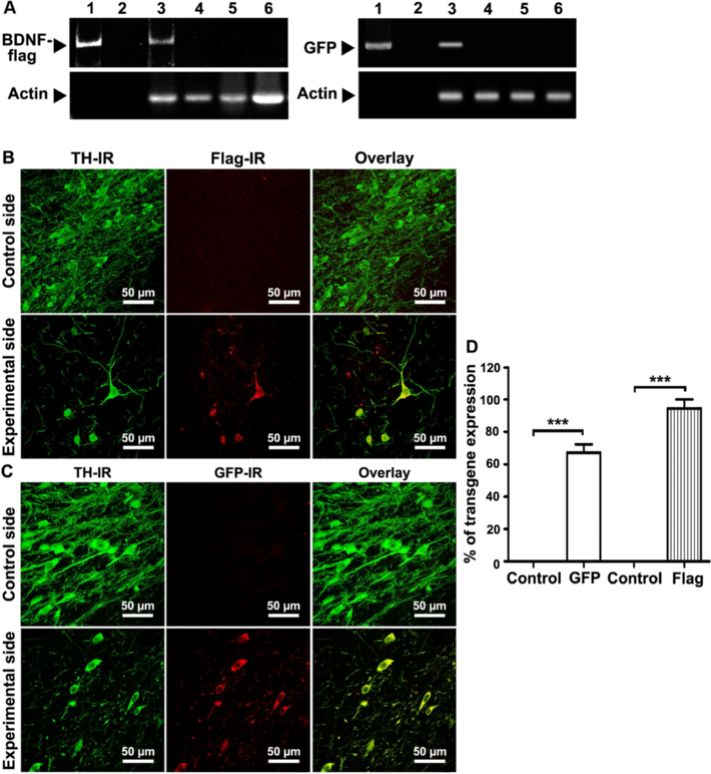
Transgenic expression in substantia nigra (SN) of hemiparkinsonian rats at week 2 after transfection using NTS-polyplex. **a** Representative photograph of RT-PCR amplicons showing BDNF-flag and GFP expression. Actin was used as a control. Lane 1, plasmid phDAT-BDNF-flag or phDAT-EGFP (positive control); lane 2, PCR reagents without cDNA (internal control); lane 3, SN (substantia nigra) with lesion and transfected with phDAT-BDNF-flag or phDAT-EGFP; lane 4, contralateral SN without lesion and transfection; lane 5, striatum ipsilateral to the side with lesion and nigral transfection; lane 6, contralateral striatum. Representative confocal micrographs showing TH-immunoreactivity (IR) and flag-IR (**b**) or green fluorescent protein (GFP)-IR (**c**) in the substantia nigra (SN) of hemiparkinsonian rats. The control side are micrographs of the intact SN contralateral to the side with the lesion and transfection (Experimental side). **d** Graph showing the percentage of flag or GFP expression with respect to the total TH+ cells. *n* = 3 rats per each experimental condition. One-way ANOVA and Bonferroni post hoc test. *** *P* < 0.0001

To discriminate the BDNF-transgene expression from the endogenous BDNF-gene product, a BDNF transgene labeled with the flag sequence was transfected with NTS-polyplex in the ipsilateral SN one week after the 6-OHDA lesion was made. Positive controls of transfections were hemiparkinsonian rats transfected with phDAT-EGFP. At week 2 after transfection, an RT-PCR analysis showed the expression of BDNF-flag or GFP mRNA in the SN with 6-OHDA lesion (Fig. 2a). On the contrary, neither the untransfected, contralateral SN nor the two striatal nuclei had BDNF-flag or GFP mRNA expression (Fig. 2a). At week 2 after transfection, a confocal microscopy analysis of double-stained mesencephalon slices showed flag immunoreactivity in 94 ± 6 % of TH+ cells of hemiparkinsonian rats that were transfected with phDAT-BDNF-flag (Fig. 2b and d). GFP immunoreactivity collocated with 67 ± 6 % of TH+ cells after transfection of phDAT-EGFP (Fig. 2c and d). In contrast, no immunoreactivity to flag or GFP was observed in the intact and untransfected SN (Fig. 2b-d).

### Structural recovery

**Fig. 3:**
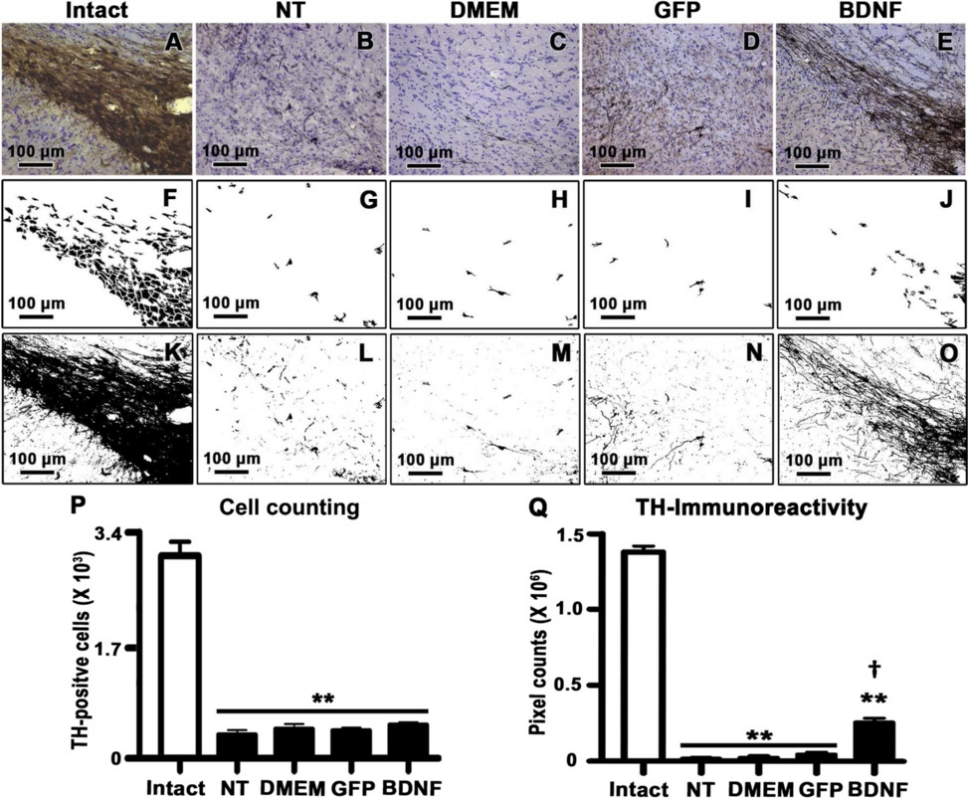
Sprouting of TH+ fibers in the substantia nigra of hemiparkinsonian rats at week 2 after transfection with the phDAT-BDNF-flag using NTS-polyplex. Representative micrographs of TH-immunohistochemistry and Cresyl Violet counterstaining (**a-e**) of the substantia nigra (SN) from hemiparkinsonian rats at three weeks after a 6-OHDA striatal lesion, and two weeks after applying one of the following treatments in the ipsilateral SN: without transfection (NT), Dulbecco’s Modified Eagle Medium injection (DMEM), phDAT-EGFP (GFP), or phDAT-BDNF-flag (BDNF) transfection. Intact refers to the SN without striatal 6-OHDA lesion. Illustrations of image processing using ImageJ software for quantification of the number of TH+ cells (**f-j**) and TH-immunoreactivity density (**k-o**). The bar graphs show the counting of TH+ cells (**p**) and TH-immunoreactivity density (**q**) in the experimental conditions as mentioned above. Values are expressed as the mean ± SE; *n* = 3 animals per group. ** *P* < 0.001 when compared with the intact side. † *P* < 0.001 when compared with the negative control groups. One-way ANOVA with Bonferroni’s post hoc test. Scale bar = 100 μm

Three weeks after the lesion, 6-OHDA decreased by 85 % the TH+ cell bodies (Fig. 3b-d, g-i, and p) and by 95 % their neuritic processes (Fig. 3b-d, l-n, and q) as compared with the intact side (Fig. 3a, f, k). Two weeks after BDNF-flag transfection (three weeks after the 6-OHDA injection), the average number of surviving TH+ cells was similar to those counted in the groups with GFP transfection, sham transfection (DMEM injection), or without transfection (NT) (Fig. 3p). Regardless, a 7-fold increase in TH+ fibers was detected within the boundaries of the SN transfected with phDAT-BDNF-flag (Fig. 3e, o, and q) as compared with the other three negative controls (Fig. 3b-d, l-n, and q).

**Fig. 4:**
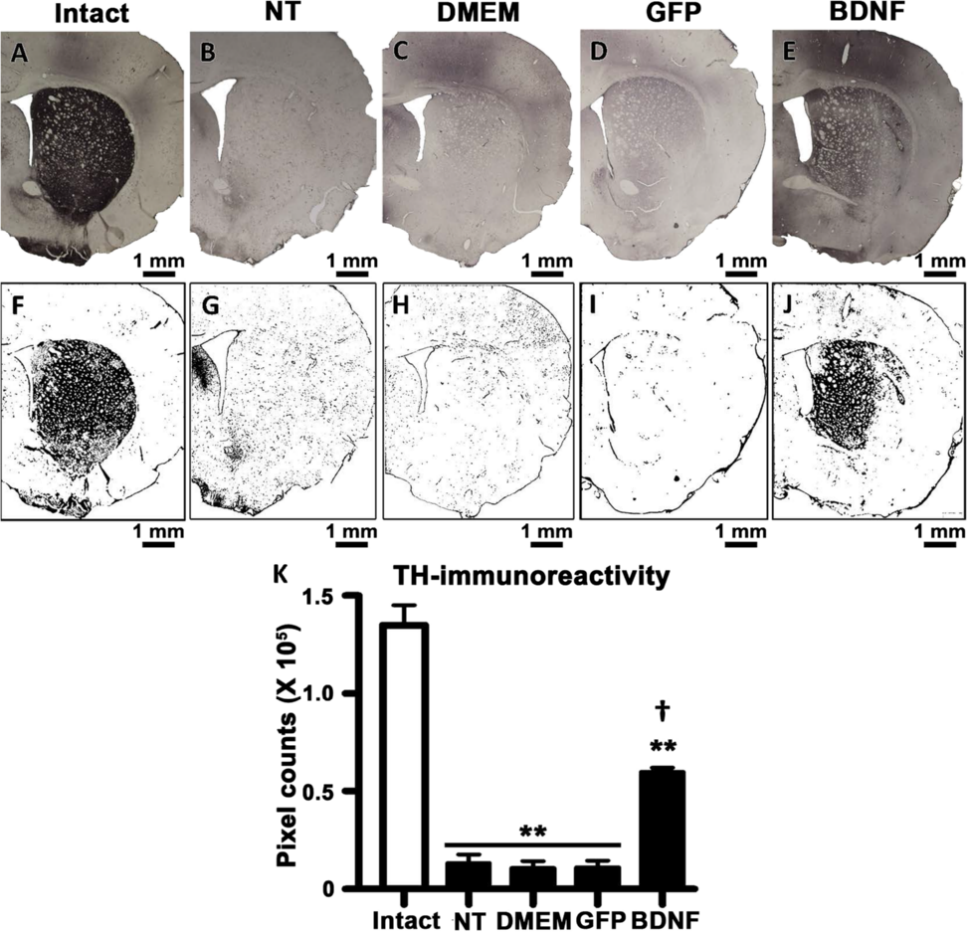
Significant recovery of TH+ fibers in the striatum of hemiparkinsonian rats at week 2 after transfection with the phDAT-BDNF-flag using NTS-polyplex. Representative micrographs of TH-immunohistochemistry of the striatum of hemiparkinsonian rats at three weeks after a 6-OHDA striatal lesion, and two weeks after applying one of the following treatments in the ipsilateral substantia nigra: without transfection (NT), Dulbecco’s Modified Eagle Medium injection (DMEM), phDAT-EGFP (GFP), or phDAT-BDNF-flag (BDNF) transfection**(a-e**. Illustrations of image processing using ImageJ software for quantification of TH-immunoreactivity density (**f-j**). The bar graph shows TH-immunoreactivity density (**k**) in the experimental conditions as mentioned above. Values are expressed as the mean ± SE; *n* = 3 animals per group. ** *P* < 0.001 when compared with the intact side. † *P* < 0.001 when compared with the negative control groups. One-way ANOVA with Bonferroni’s post hoc test

In the striatum, the 6-OHDA injection reduced by 90 ± 3 % the TH+ axon density, which was not modified by GFP transfection or DMEM injection (Fig. 4b-d, g-i and k). On the contrary, a 5-fold increase of TH+ fibers in the striatum of hemiparkinsonian rats was detected when compared with the other three negative controls at two weeks after phDAT-BDNF-flag transfection using NTS-polyplex (Fig. 4e, j and k).

### Dopamine level recovery

**Fig. 5:**
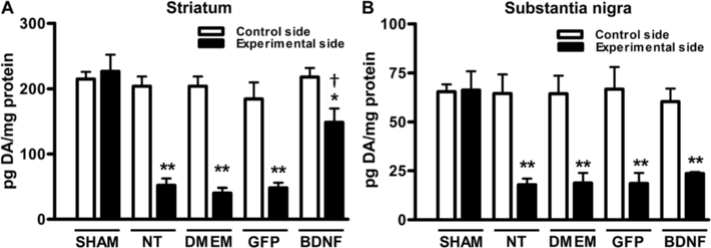
Partial recovery of striatal DA levels at week 2 after transfection with the BDNF-flag gene into DA neurons using NTS-polyplex. DA levels were measured using HPLC with electrochemical detection in the striatum (**a**) and in the substantia nigra (**b**) at three weeks after unilateral microinjection of 6-OHDA in one striatum (experimental side), and at two weeks after applying one of the following treatments in the ipsilateral substantia nigra: without transfection (NT), Dulbecco’s Modified Eagle Medium injection (DMEM), phDAT-EGFP (GFP), or phDAT-BDNF-flag (BDNF) transfection. The SHAM lesion group was injected in the striatum only with PBS (6-OHDA vehicle). The control side refers to the intact tissues (striatum or substantia nigra) contralateral to the side with the lesion and transfection. The experimental side refers to tissues ipsilateral to the lesion and transfection. Values are expressed as the mean ± SE; *n* = 4 animals per group. * *P* < 0.05, ** *P* < 0.01 when compared with the values caused by a sham lesion. † *P* < 0.05 when compared with the values of the untransfected side (NT). One-way ANOVA with Bonferroni’s post hoc test

Three weeks after the 6-OHDA lesion, a significant 70 % decrease of dopamine levels was measured in the striatum and SN when compared with those in the intact, contralateral side (Fig. 5a, b). Notably, two weeks after transfecting the SN with the phDAT-BDNF-flag a 75 % recovery of striatal DA levels was observed in comparison with the intact, contralateral striatum (Fig. 5a). DA levels in the striatum was 5-fold higher than those in the side with lesion and without transfection (NT), sham transfection (DMEM injection), or GFP transfection (Fig. 5a). However, no significant recovery of DA levels occurred in the SN transfected with the phDAT-BDNF-flag in comparison with the other control groups (Fig. 5b).

### Motor behavior recovery

**Fig. 6:**
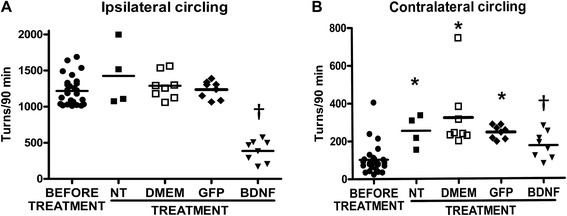
Drug induced-circling behavior in hemiparkinsonian rats at two weeks after transfecting the substantia nigra using NTS-polyplex. One week after a 6-OHDA striatal injection treatments were applied into the substantia nigra (arrow) as follows; without transfection, Dulbecco’s Modified Eagle Medium injection (DMEM), phDAT-EGFP or phDAT-BDNF-flag transfection. Ipsilateral circling behavior was included by amphetamine (8 mg/Kg, i.p.), whereas contralateral circling behavior was induced by apomorphine (0.5 mg/kg, s.c.) at weeks 1 (before treatment; *n* =28 animals) and 3 weeks after the striatal 6-OHDA lesion; *n* = 4 for the NT group and *n* = 8 animals for the rest of the groups. * *P* < 0.05, when compared with the values measured at week 1. † *P* < 0.05 when compared individually with the values of the other groups at week 3. Kruskal-Wallis test with Dunns post hoc test

Hemiparkinsonian rats exhibiting equivalent values of circling behavior in 90 min (1216 ± 143 ipsilateral turns, Mean ± SE, induced by methamphetamine and 103 ± 15 contralateral turns, Mean ± SE, induced by apomorphine) received different treatments into the substantia nigra one week after the ipsilateral-striatal 6-OHDA injection. Two weeks after the lesion, the amphetamine-induced circling behavior of negative control rats (NT, DMEM and GFP) did not show statistical differences when compared with that at week 1 after lesion (Fig. 6a). Whereas, the apomorphine-induced circling behavior significantly increased by more than 198 % in the groups without transfection and in those with sham or GFP transfections, suggesting hypersensitivity of dopamine receptors (Fig. 6b). A significant reduction of both methamphetamine- and apomorphine-induced circling behaviors occurred with transfection of phDAT-BDNF-flag (Fig. 6). When compared with the circling behavior of control hemiparkinsonian rats, the reduction in methamphetamine-induced rotational behavior was 68 % at week 2 after phDAT-BDNF-flag transfection, whereas the reduction was 30 % for apomorphine-induced rotational behavior at the same time (Fig. 6).

**Fig. 7:**
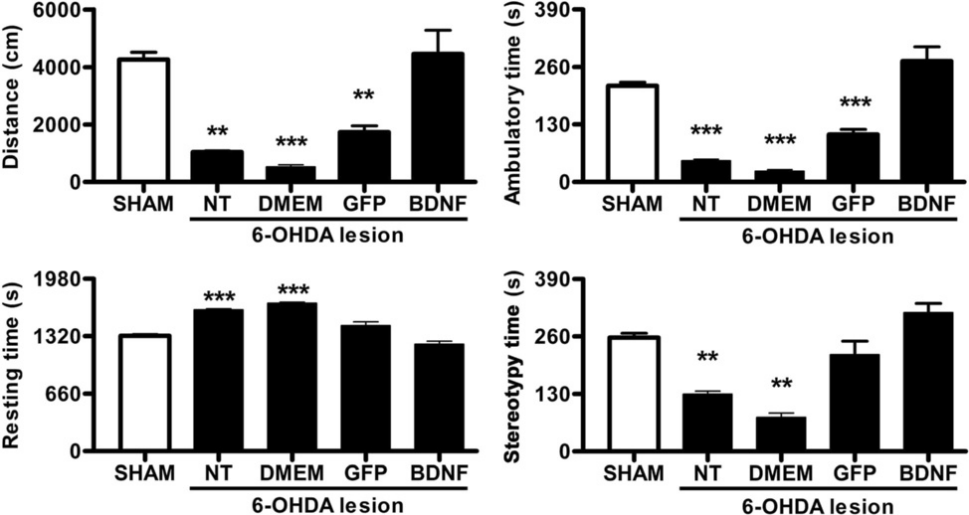
Spontaneous motor behavior of hemiparkinsonian rats in an open field at two weeks after transfecting the substantia nigra using NTS-polyplex. SHAM refers to the group of rats with a false lesion. One week after a 6-OHDA striatal injection treatments were applied into the substantia nigra as follows: without transfection (NT), injection with Dulbecco’s Modified Eagle Medium (DMEM), and transfection with phDAT-EGFP (GFP) or phDAT-BDNF-flag (BDNF). Values are expressed as the mean ± SE; *n* = 8 animals per group. ** *P* < 0.01, *** *P* < 0.001 vs. SHAM. One-way ANOVA with Bonferroni’s post hoc test

The spontaneous motor behavior of hemiparkinsonian rats in an open-field test also showed recovery at two weeks after the phDAT-BDNF-flag transfection. Accordingly, the values of the four variables (distance traveled, ambulatory time, resting time, and stereotypy time) measured in hemiparkinsonian rats with BDNF-flag transfection were not significantly different from those recorded in rats with a false lesion (Fig. 7). In contrast, the values of distance traveled, ambulatory time and stereotypy time in the groups without transfection or DMEM injection (sham transfection) were significantly lower than those registered in rats with a false lesion were. In the group with GFP transfection, only the distance traveled and ambulatory time showed significant decreases, whereas stereotypy time did not differ from that measured in rats with a sham lesion. The values of resting time showed a significant increase only in hemiparkinsonian rats without transfection or with sham transfection in comparison with the values recorded in animals with a sham lesion (Fig. 7).

## Discussion

Our results show for the first time that the overexpression of BDNF within the DA neurons of the SN ameliorates the acute hemiparkinsonism induced by a striatal injection of 6-OHDA in the rat. The ability of NTS-polyplex to transfect genes into surviving DA neurons [29, 45] was a key factor to increase their levels of BDNF and thus strengthen its neurotrophic effects on the injured nigrostriatal system. Our results showed that the residual DA neurons of the SN still maintain the expression of NTSR1 and its ability to mediate the transfer of GFP and BDNF-flag gene by NTS-polyplex. These results agree with the notion that NTS-polyplex enters DA neurons through NTSR1 internalization to deliver its genetic cargo to the cell nucleus and express the transgenic protein [21, 24, 27–29]. The expression of NTSR1 is not only maintained in experimental neurodegeneration [46, 47], but also in PD patients in whom the presence of NTSR1 mRNA and protein has been clearly demonstrated by ligand binding [30], autoradiography [31], and in situ hybridization [32]. The presence of NTSR1 in residual DA neurons opens up the possibility of using the NTS-polyplex-mediated BDNF gene delivery as a therapy for PD.

The assertion that BDNF influences DA neurons through autocrine and paracrine mechanisms has been sustained by the finding that those neurons coexpress BDNF and its TrkB receptor [1, 2]. In PD, the decreasing levels of BDNF and TrkB accompanying the progressive death of DA neurons inevitably lead to the loss of their own neurotrophic source to halt the progress of neurodegeneration [9, 11]. Our results in an experimental rat model of PD showed that strengthening of the neurotrophic source by BDNF transfection into surviving DA neurons was able to partially rescue the functional innervation of the nigrostriatal system. BDNF-flag expression caused a significant sprouting of DA fibers and increased DA levels in the injured striatum as soon as two weeks after transfection. Although significant sprouting of DA fibers was also seen in the SN, the number of DA neurons and DA levels in this nucleus were not increased, suggesting that BDNF-flag expression does not stimulate neurogenesis but promotes neuritogenesis in the SN and striatum. These results support the inability of BDNF to enhance neurogenesis in adult mice and rats [48] and its ability to stimulate axonal elongation and dendritic branching in cell cultures and in experimental animals [49–53]. Our results about the lack of neurogenesis and the stimulation of neuritogenesis also agree with previous studies in which a BDNF gene was transduced by an AAV vector in the SN [20] or BDNF protein was provided by epigenetically induced BDNF-secreting human mesenchymal stem cell grafts [19]. Regardless, the partial recovery of striatal DA innervation induced by BDNF-flag expression led to substantial reversal of drug-induced motor asymmetry in hemiparkinsonian rats. Even more striking, BDNF-flag expression completely corrected the impairment of spontaneous motor behaviors in hemiparkinsonian rats. Those results indicate that the residual DA neurons keep the responsiveness to BDNF and support the presence of TrkB receptors on these neurons as recently shown [19]. Because TrkB mRNA expression has been shown in the SN of PD patients, BDNF-gene therapy might be effective in the early stage of PD [10]. Clinical studies show that the intensive rehabilitation treatment increases the BDNF levels and improves PD signs in patients in the early stages of the disease [54], and that there is a positive correlation between advanced stages of the disease and serum levels of BDNF, may be as a potential compensatory mechanism [55]. These results suggest that BDNF treatment might be effective at any stage of the disease. However, the use of other animal models different from 6-OHDA or implementing chronic models treated with BDNF is necessary to prove the feasibility of BDNF as a treatment for PD.

Preclinical studies with GDNF [29, 56, 57], neurturin [58], or BDNF as reported and shown in this study have allowed gaining insight into the potential benefits of gene therapy to restore the DA innervation in PD. However, this procedure has some limitations. For example, those expressing GDNF have been associated with undesirable behavioral effects resulting from aberrant innervation of the regenerating DA fibers [59] and the decrease of DA synthesis [60]. Others have documented a striatal DA hypofunction after a chronic intranigral administration of BDNF [61]. These biosafety concerns urge for more research devoted to identify the best vectors and genes that provide the maximum benefits with the minimum of adverse effects. In addition, the regulation of neurotrophic gene expression must be implemented in the gene vectors in order to switch off the gene expression in the case of development of unexpected undesirable effects.

Although viral gene therapy for PD has been under evaluation for a number of years [16, 62, 63], the availability of alternate gene transfer methods remains important considering the concerns with potential immune reactions to viral vectors [64, 65] and potential oncogenicity of viral vectors able to integrate the transgene into the host genome [66, 67]. A recent study supports the lack of inflammatory effect of systemically or locally injected NTS-polyplex [22]. Moreover, the transfer of the cerebral dopamine neurotrophic factor (CDNF) into DA neurons via NTS-polyplex did not induce neuroinflammation in vivo and additionally exerted an anti-inflammatory effect in the 6-OHDA rat model of PD [45]. These results show the safety of local injection of NTS-polyplex NPs and sustain its possible application in the clinic. Recent studies in PD patients demonstrate that the surgical approach to SN is a feasible and safe procedure for viral gene transfer in humans [68]. Nevertheless, invasive injection in the SN is the major limitation of NTS-polyplex because is unable to cross the blood–brain barrier (BBB) when injected intravenously [69]. To overcome this limitation, two methods of safe administration can be used. The most promising method is the reversible opening of the BBB using focused ultrasound (FUS) that allows the targeted delivery of nanoparticles in the order of micrometers in diameter [70], and by their size (50–150 nm) [22–24], the NTS polyplex nanoparticles can pass through the transient opening of the BBB. The other method is the intrathecal administration [71], which might allow the targeted gene delivery of NTS-polyplex through the cerebrospinal fluid. This application is feasible because NTS-polyplex enables targeted gene delivery into cancer cells through the blood stream as previously demonstrated in in neurobalstoma and breast cancer animal models [23, 69].

## Conclusions

Our results show that in a 6-OHDA-induced early hemiparkinsonism, surviving DA neurons conserve the expression of NTSR1 on cell bodies and neurites that enabled gene transfection by NTS- polyplex. The expressed BDNF-flag produced partial biochemical, structural, and behavioral recovery from early parkinsonism. The major effect of BDNF-flag was on the DA branching in the striatum and SN. The lack of effect on the cell number might be accounted by the inability of BDNF to stimulate subventricular zone neurogenesis in adult mice and rats [48]. Regardless, BDNF gene therapy can be associated to other neurogenesis-stimulating treatment to reinforce the sprouting of new DA terminals and provide maintenance of their synaptic connectivity. The results of our work and the success of preclinical studies with other neurotrophic genes strongly support the feasibility of using the NTS-polyplex in gene therapy for an efficient and selective recovery of striatal DA innervation in the early stages of PD [50].

## Abbreviations

6-OHDA: 6-hydroxydopamine
AAV: Adeno-associated virus
BDNF: Brain-derived neurotrophic factor
bp: Base pair
DAT: Dopamine transporter
DMEM: Dulbecco’s modified eagle medium
EGFP: Enhanced green fluorescent protein
FITC: Fluorescein isothiocyanate
GDNF: Glial-cell derived neurotrophic factor
kbp: Kilo base pair
KP: Karyophilic peptide
NTS: Neurotensin
NTSR1: Neurotensin receptor type 1
PD: Parkinson’s disease
SN: Substantia nigra
TRITC: Tetramethylrhodamine
TrkB: Tropomyosin related kinase B
